# Comparative analysis of retrograde intrarenal surgery and modified ultra-mini percutaneous nephrolithotomy in management of lower pole renal stones (1.5–3.5 cm)

**DOI:** 10.1186/s12894-020-00586-6

**Published:** 2020-03-16

**Authors:** Zhuohang Li, Cong Lai, Arvind K. Shah, Weibin Xie, Cheng Liu, Li Huang, Kuiqing Li, Hao Yu, Kewei Xu

**Affiliations:** 1 Department of Urology, Sun Yat-sen Memorial Hospital, Sun Yat-sen University, 107 West Yanjiang Road, Guangzhou, 510210 China; 2 Department of Urology, Bir Hospital, National Academy of Medical Sciences, Kathmandu, Nepal

**Keywords:** Lower pole stones, Modified ultra-mini percutaneous nephrolithotomy, Semi-supine combined lithotomy position, Retrograde intrarenal surgery

## Abstract

**Background:**

To compare the safety and efficacy of retrograde intrarenal surgery (RIRS) and modified Ultra-mini percutaneous nephrolithotomy (UMP) in semi-supine combined lithotomy position for the management of 1.5–3.5 cm lower pole renal stones (LPSs).

**Methods:**

A total of 63 patients with 1.5–3.5 cm LPSs who underwent RIRS (*n* = 33) or modified UMP (*n* = 30) in diameter between January 2017 and January 2019 were analyzed retrospectively. Modified UMP was performed in semi-supine combined lithotomy position and a 9.5/11.5 F ureteral access sheath (UAS) was inserted during the procedure in order to maintain low pelvic pressure and to facilitate the removal of stone fragments. Base-line parameters, stone characteristics, illness condition, operation time, postoperative hemoglobin (Hb) drop, postoperative creatinine (Cr) elevation, length of hospital stay, length of postoperative hospital stay, stone-free rate (SFR) and complications were compared between the two groups.

**Results:**

There were no significant differences between the two groups in base-line parameters, stone characteristics and illness condition. The mean operating time of RIRS group was longer than UMP group (95.61 ± 21.9 vs. 55.0 ± 16.1 min, *p* < 0.001). The mean postoperative Hb drop was less in RIRS group (7.42 ± 4.7 vs. 15.70 ± 9.8 g/L, *p* < 0.001). The length of hospital stay and postoperative hospital stay for RIRS were shorter than UMP (4.76 ± 1.1 vs. 5.83 ± 0.8 d, *p* < 0.001, 2.97 ± 0.9 vs. 4.07 ± 0.9 d, *p* < 0.001). The Early SFR was higher in UMP group (54.5 vs. 80.0%, *p* < 0.050) while SFR at 1-month and 3-months postoperatively was similar in both groups (*p* = 0.504, *p* = 0.675). There were no significant differences between the two groups in complications (*p* = 0.228).

**Conclusion:**

For patients with 1.5–3.5 cm LPSs, both modified UMP and RIRS are safe and viable. The modified UMP technique was used in this study, application semi-supine combined lithotomy position and the retention of UAS can improve the surgical efficiency and maintain low pressure perfusion in the kidney, which resulted in superior treatment efficacy. Therefore, we highly recommend this technique for LPSs with heavy stone burdens.

## Background

Due to the anatomic characteristics of the lower calyx, LPSs are difficult to be eliminated through the ureter, even if the stones had been fragmented [1, 2]. RIRS can be used to deal with LPSs of 1.0–2.0 cm, while Percutaneous nephrolithotripsy (PCNL) is mainly used to deal with LPSs with larger diameter or when RIRS failed to resolve the stone. With the growing advancement of medical devices and technologies, the application of RIRS has gradually expanded to kidney stones with a size of more than 2.0 cm or even more than 3.0 cm [3, 4]. On the other hand, many emerging miniaturized PCNL technologies, including UMP, which can reduce the risk of kidney injury after surgery, are also increasingly used for the treatment of stones [5–7]. RIRS and UMP both have their own advantages and disadvantages leading to controversies regarding the use of these surgical techniques to treat LPSs.

RIRS is performed through natural orifice. It can reduce hospitalization time and the risk of bleeding. Previous studies indicated that although the safety is more guaranteed, the SFR of RIRS may not be so effective in the treatment of stones larger than 2.0 cm [3]. However, owing to the accumulation of ureteroscopic surgery experience and the rapid improvement of ureteroscope apparatus, the clinical application of RIRS is more extensive. It has been reported that the SFR can reach 73.6% when RIRS is used to treat the 2.0–3.5 cm kidney stones and 90.2% when used to treat LPSs with a diameter of 1.5–2.5 cm [3, 8].

Compared to RIRS, PCNL could reach a higher SFR though it presents greater surgical risks. In 2013, Janak Desai developed the Ultra-mini percutaneous nephrolithotomy (UMP) with a tract size of 11–14 F in order to reduce the risk of complications [9]. As the percutaneous tract becomes smaller, the operation efficiency decreases and the intra-renal pressure may soar too high during the procedure. Therefore, it has been reported that the best indication of UMP is for kidney stones less than 2 cm [10]. We have previously reported a modified UMP technique where operation was performed under the semi-supine combined lithotomy position with the presence of the ureter access sheath (UAS) to increase perfusion while maintain low intrapelvic pressure. We have demonstrated that this technique could be used to treat renal calculi within 3.0 cm with good safety, while achieving 90.9% of the primary stone clearance rate and 100% of the SFR after auxiliary treatment [11].

Previous studies have shown that UMP and RIRS are both safe and effective in the treatment of medium-sized (1.0–2.0 cm) urinary calculi, the SRF immediately after surgery was high (UMP, 84%; RIRS, 87%) [12]. Zhang et al. reported that the 3-months SRF of UMP and RIRS in the treatment of 1.0–2.0 cm LPSs was 98 and 92%, respectively [13]; while SFR for these methods in the treatment of 1.0–3.5 cm renal stones was 92 and 96%, respectively [14]. However, the outcomes of UMP and RIRS in the treatment of large LPSs remains inconclusive.

In this study, we analyzed two groups of large LPSs (1.5–3.5 cm) cases who underwent modified UMP or RIRS in our hospital to compare the efficacy and safety of the two techniques. We hope our results could provide some clinical evidence for urologists in choosing surgical options for 1.5–3.5 cm LPSs.

## Methods

This is a retrospective analysis of patients treated between January 2017 and January 2019 in department of Urology, Sun Yat-sen Memorial Hospital, approved by the hospital ethics committee. Informed consents were signed by all patients. Patients aged between 18 and 80 years with single LPS of 1.5–3.5 cm were included in this study. Stone volume were calculated using the equation: length × width × height × π/6 [15]. Exclusion criteria are as follows: patients with renal malignancy, ectopic kidney, transplanted kidney stone, spongy kidney, polycystic kidney and uncontrolled pyonephrosis. All included patients were thoroughly evaluated by medical history, physical examination, blood and urine routine tests, urine culture, blood biochemistry and other laboratory tests. Intravenous urography (IVU) and urinary system CT examination were conducted. LPSs were diagnosed by IVU and CT plain scan. The size of the stone was measured by the long axis. Ultrasonography was used to evaluate the presence of hydronephrosis. The patients were divided into two groups depending on the treatment method, RIRS group (*n* = 33) and UMP group (*n* = 30). Blood routine and serum biochemical tests were performed in all patients after surgery. Plain abdominal radiograph of the kidney, ureter and bladder (KUB) were performed on 24–48 h, 1-month and 3-months after surgery to evaluate the early SFR, 1 month SRF and 3 months SRF, respectively. Residual fragments less than 2 mm were considered “stone-free”.

### RIRS techniques

For RIRS procedure, patients were put in lithotomy position under epidural anesthesia. Ureteroscope was performed to insert a guidewire (Boston Scientific®) into the pelvis. The guide-wire was inserted and a 12/14 F or 9.5/11.5 F ureter access sheath (UAS) (COOK®) was placed through it into the proximal ureter. Then a detachable flexible ureteroscope (POLY DIANOST®) was used to examine the collection system and locate to the LPSs. The stones that were mobile were repositioned into the upper or middle calyx, and those that were immobile are treated with laser lithotripsy in situ. Holmium: YAG laser (Lumenis®) was set at low energy and high frequency (0.6–1.2 J, 20–30 Hz) for lithotripsy. Large fragments were removed by a nitinol basket. Prior to the completion of the operation, a 6F JJ stent was placed.

### Modified UMP techniques

In the modified UMP group, patients were put in the semi-supine combined lithotomy position with operating side elevated at 45° under epidural anesthesia. A 9.5/11.5 F UAS was inserted using the same protocol as the RIRS group. 30–50 ml of saline was injected through the UAS to obtain artificial hydronephrosis. Percutaneous renal puncture was performed under the guidance of ultrasonography. Immediately after that, 10 F and 14 F fascia dilators were used sequentially to establish the tract. After the placement of the 13 F sheath, UMP was performed by holmium laser lithotripsy (1.0–2.0 J, 20–30 Hz). Stone fragments were washed out by an irrigation pump. The UAS was used as an outflow tract for irrigation fluid along with stone fragments. After lithotripsy, ultrasonography was performed again to check for residual stones. The retention of nephrostomy tube was determined according to the removal of stone fragments and bleeding of the tract at the end of surgery. All patients received a 6F JJ stent. The nephrostomy tube was removed between 24 and 48 h after the operation. The operations in both groups were performed by Professor Kewei Xu. The JJ stent was removed around 2–4 weeks in both groups.

### Statistical analysis

Data analysis was performed on SPSS 23.0 software. Continuous data was presented as mean ± SD, and the Student’s t-test or rank sum test was used to analyze the differences. Dichotomous data was analyzed by the chi-square or Fisher’s exact tests. *p* < 0.05 was considered statistically significant.

## Results

There were no statistically significant differences between RIRS group and UMP group in mean age (49.12 ± 11.5 vs. 52.50 ± 11.2 years, *p* = 0.242), gender (*p* = 0.824), mean body mass index (24.20 ± 2.9 vs. 23.45 ± 2.7 kg/m^2^, *p* = 0.289), mean stone length (2.57 ± 0.5 vs. 2.68 ± 0.5 cm, *p* = 0.377), mean stone volume (5.47 ± 2.00 vs. 5.54 ± 2.63 cm^3^, *p* = 0.904), stone side (*p* = 0.479), comorbidities (*p* = 0.894), degree of hydronephrosis (*p* = 0.740) and preoperative urine WBC (*p* = 0.246) (Table 1). Three of the patients had positive urine culture before surgery and received anti-infection treatment for two weeks prior to surgery.

**Table 1:** Comparison of the baseline characteristics of included patients

	RIRS	UMP	*P*
No. patients	33	30	
Male/female *n*(%)	20/13 (60.6%/39.4%)	19/11 (61.9%/38.1%)	0.824
Mean age (years)	49.12 ± 11.5 (26–77)	52.50 ± 11.2 (22–70)	0.242
Mean BMI (kg/m^2^)	24.20 ± 2.9 (19.11–30.39)	23.45 ± 2.7 (16.44–27.11)	0.289
Mean stone length (cm)	2.57 ± 0.5 (1.5–3.3)	2.68 ± 0.5 (1.6–3.5)	0.377
Mean stone volume (cm^3^)	5.47 ± 2.00 (1.4–8.3)	5.54 ± 2.63 (2.0–9.8)	0.904
Stone side *n* (%)			0.479
Left	18 (54.5%)	19 (63.3%)	
Right	15 (45.5%)	11 (36.7%)	
Comorbidities *n* (%)			0.894
None	20 (60.6%)	18 (60.0%)	
Hypertension	5 (15.2%)	5 (16.7%)	
Diabetes	5 (15.2%)	3 (10.0%)	
Others	3 (9.1%)	4 (13.3%)	
Hydronephrosis *n* (%)			0.740
0 degree	20 (60.6%)	15 (50.0%)	
1 degree	9 (27.3%)	9 (30.0%)	
2 degree	2 (6.1%)	4 (13.3%)	
3 degree	2 (6.1%)	2 (6.7%)	
Preoperative urine WBC *n* (%)			0.246
–	14 (42.4%)	14 (46.7%)	
+	6 (18.2%)	10 (33.3%)	
++	4 (12.1%)	3 (10。0%)	
+++	9 (27.3%)	3 (10.0%)	

In the RIRS group, 87.87% (29/33) of patients used 12/14 F ureter access sheath and the remaining patients switched to 9.5/11.5 F UAS due to difficulty in placement. In the UMP group, a single percutaneous tract (13 F) was established for lithotripsy and stone removal. Nephrostomy tube were indwelled in 53.3% (16/30) of patients in the UMP group. There were no intraoperative complications or changes of surgical methods in the two groups. UAS was successfully implanted in both groups, and no patient was excluded from the study because of unsuccessful use of UAS. The mean operation time of RIRS group was longer than UMP group (95.61 ± 21.9 vs. 55.0 ± 16.1 min, *p* < 0.001). The mean postoperative Hb decline of the UMP group was significant higher (RIRS: 7.42 ± 4.7 vs. UMP: 15.70 ± 9.8 g/L, *p* < 0.001). The mean postoperative Cr elevation was 7.88 ± 9.1 mg/dl in RIRS group and 11.40 ± 13.5 mg/dl in UMP group (*p* = 0. 235). The mean postoperative hospital stay of RIRS group was shorter than that of UMP group (2.97 ± 0.9 vs. 4.07 ± 0.9 d, *p*< 0.001). The mean hospitalization time of RIRS group was shorter than that of UMP group (4.76 ± 1.1 vs. 5.83 ± 0.8 d, *p*< 0.001). (Table 2).

**Table 2:** Comparison of the curative effect of the two groups of patients

	RIRS	UMP	*P*
No. patients	33	30	
Mean operation time (min)	95.61 ± 21.9 (35–145)	55.0 ± 16.1 (30–95)	<0.001
Mean Hb drop (g/L)	7.42 ± 4.7 (1–22)	15.70 ± 9.8 (0–41)	<0.001
Mean Cr elevation (mg/dl)	7.88 ± 9.1 (0–49)	11.40 ± 13.5 (0–63)	0.235
Hospital stay (d)	4.76 ± 1.1 (3–8)	5.83 ± 0.8 (4–8)	<0.001
Postoperative hospital stay (d)	2.97 ± 0.9 (2–6)	4.07 ± 0.9 (2–6)	<0.001
Early SFR, *n* (%)	18 (54.5%)	24 (80.0%)	<0.050
1 month SFR, *n* (%)	28 (84.8%)	28 (93.3%)	0.504
3 month SFR, *n* (%)	30 (90.9%)	29 (96.7%)	0.675
Post-op complications, *n* (%)			0.228
Fever (Clavien I)	4 (12.1%)	3 (10.0%)	
Hematuria (Clavien I)	0	2 (6.7%)	
Urosepsis (ClavienII)	1 (3.0%)	0	

In terms of complications, there were four cases of postoperative fever (Clavien I) in RIRS group and three cases in UMP; and all patients improved after being treated with antipyretic drugs. One case of postoperative urosepsis (ClavienII) were presented in RIRS group and showed improvement after treatment. Two patients presented postoperative gross hematuria (Clavien I) in UMP group and were spontaneously relieved after 48 h. Patients in UMP group did not have complications such as blood transfusion, interventional embolization, colon injury and pleural injury. The difference in complications between the two groups was not statistically significant (*p* = 0.228). SFR in UMP group was higher than RIRS group at all points, early SFR (80.0% vs. 54.5%, *p* < 0.001), 1-month SFR (93.3% vs. 84.8%, *p* = 0.504) and 3-month SFR (96.7% vs. 90.9%, *p* = 0.675) although the latter two groups did not present statistical significance (Table 2). In the UMP group, one case of remaining stones was excreted after extracorporeal shock wave lithotripsy (ESWL). There were three cases of residual stones in the RIRS group, two cases were treated with ESWL, and RIRS was re-performed in one case. The final SFR of both groups was 100%.

## Discussion

The treatment of lower pole renal calculi is a difficult point in urology. As the anatomical structure of the lower calyceal is not conducive for stone excretion, ESWL is less effective in the treatment of LPSs [1, 16, 17]. Some surgeons believed that either RIRS or PCNL surgery is needed even if the diameter of LPSs is less than 1.0 cm [18]. Endourology surgery is widely used to treat calculi with diameter less than 2.0 cm [19]. The application of RIRS or UMP in the treatment of calculi larger than 2 cm in diameter is rarely reported.

RIRS has developed rapidly in recent years and its indications for the treatment of kidney stones are becoming more extensive. It has been reported that SFR of kidney stones over 2.0 cm treated with RIRS is about 66.7–94.1% [20]. However, flexible ureteroscope still has its own drawbacks, including instrument damage and iatrogenic infection [21–23]. In this study, disposable and detachable flexible ureteroscope was used to avoid those problems. The lower calyx calculi were moved to the renal pelvis or the upper calyx by a stone basket to facilitate the operation of lithotripsy. However, due to the low efficiency of RIRS, the operation time is linearly correlated with the stone volume, which could lead to the increase in the incidence of postoperative fever and urosepsis [24, 25]. In our study, the mean operative time was 95.61 ± 21.9 min, with postoperative fever in four cases and sepsis in one case. However, none of the four patients with fever after operation had used antipyretics, and their body temperature returned to normal after non-drug treatment. Furthermore, when RIRS was used to treat large renal stones, SFR was low and the possibility of subsequent treatment or staging surgery was increased. In this study, SFR at 1-month after surgery was 84.8%, and SFR at 3-months after surgery was 90.9%, with three patients needed follow-up treatment. Although PCNL has the advantages of high lithotripsy efficiency and freedom from anatomical factors, its complication rate is also higher than RIRS [26]. The most common complications are bleeding and infection. Tract size is the main factor that affect bleeding in PCNL. In order to improve surgical safety, UMP was then invented in 2013 by Desai et al. [9, 10]. They reported 61 cases who underwent lithotripsy using a 6 F nephroscope through a 11–13 F PCN tract, the results showed great efficacy and safety. However, studies have shown that the decrease of PCN tract size may lead to lower perfusion efficiency and incomplete fragmentation of the stones. These drawbacks could result in prolonged operative time and increased intrapelvic pressure, which could higher the risk of postoperative infection [26–28]. Therefore, to date, that the best indication of UMP is kidney stones less than 2.0 cm and its efficiency seems to be equivalent to RIRS [10, 29].

Our research group has reported a modified UMP technique [11]. Patients were placed in semi-supine combined lithotomy position and a UAS sheath was indwelled, which improved drainage efficiency and reduced intrapelvic pressure. The results indicated that this improved surgical method for the treatment of 2.0–3.0 cm kidney stones had good efficacy and safety. Intraoperative intrapelvic pressure was stable at 5–10 mmHg, lower than the urine reflux threshold (30 mmHg). In this study, we adopted this modified UMP technique to treat 1.5–3.5 cm LPSs. The results showed that the operation time of UMP was significantly less than RIRS. The SFR of UMP group reached 96.7% and the incidence of complications was similar between the two groups. However, our data showed that the decrease of Hb and the incidence of postoperative hematuria in UMP group were higher than that in RIRS group and the postoperative hospitalization time in UMP group was also longer. In conclusion, UMP is more effective than RIRS, along with higher bleeding risk and longer hospital stay. Comparing with the other study by Wilhelm K et al. [14] focusing on UMP and RIRS for 10–35 mm renal calculi, our results of UMP showed better efficacy. This may be due to our modified technique. The semi-supine position could avoid position change during the procedure, and the UAS could provide better drainage.

In terms of postoperative catheterization, we adopted a conservative strategy for both groups. There was no indwelling JJ stent in both groups before the operation. Considering that the ureteral injury was relatively large during the operation, JJ stent was routinely indwelling for 2–4 weeks after the operation. The retention of nephrostomy tube was determined according to the intraoperative removal of stone fragments and bleeding in UMP group. The purpose of indwelling nephrostomy tube was to observe the characteristics of drainage fluid. Studies have shown that tubeless can be used in UMP to speed up its postoperative recovery, which warrants further study. During our modified UMP approach, the ureter was manipulated, which made it difficult to perform ‘totally-tubeless’ compared with traditional procedure. This could be a minor drawback in order to reach higher efficacy.

## Conclusion

In conclusion, both UMP and RIRS are safe and effective for the treatment of 1.5–3.5 cm lower pole renal calculi. The modified UMP technique could reach shorter operation time along with a higher Hb drop and longer hospital stay compared with RIRS. Future prospective studies with larger sample sizes were needed to verify and extend the findings of this study.

## Data Availability

The datasets supporting the conclusions of this article are available in the Sun Yat-sen Memorial Hospital Medical Records Room data base (Guangzhou, Guangdong, China) repository. In additional, the datasets analyzed during the current study is available from the corresponding author on reasonable request.

## Abbreviations

Cr: Creatinine
CT: Computed tomography
EAU: European society of urology
ESWL: Extracorporeal shockwave lithotripsy
GMSV: Galdakao-modified supine Valdivia position
Hb: Hemoglobin
IVU: Intravenous urography
KUB: Examinograph of the kidney, ureter and bladder
LPSs: Lower pole renal stones
PCNL: Percutaneous nephrolithotomy
RIRS: Retrograde intrarenal surgery
SFR: Stone-free rate
UAS: Ureteral access sheath
UMP: Ultra-mini percutaneous nephrolithotomy
